# Data set of the protein expression profiles of Luminal A, Claudin-low and overexpressing HER2^+^ breast cancer cell lines by iTRAQ labelling and tandem mass spectrometry

**DOI:** 10.1016/j.dib.2015.04.025

**Published:** 2015-06-17

**Authors:** Karla Grisel Calderón-González, Ma Luz Valero Rustarazo, Maria Luisa Labra-Barrios, César Isaac Bazán-Méndez, Alejandra Tavera-Tapia, Marí;aEsther Herrera-Aguirre, Manuel M. Sánchez del Pino, José Luis Gallegos-Pérez, Humberto González-Márquez, Jose Manuel Hernández-Hernández, Gloria León-Ávila, Sergio Rodríguez-Cuevas, Fernando Guisa-Hohenstein, Juan Pedro Luna-Arias

**Affiliations:** a Doctorado en Ciencias Biológicas y de la Salud, Universidad Autónoma Metropolitana, Unidad Iztapalapa, Av. San Rafael Atlixco No. 186, Col. Vicentina, Iztapalapa, C.P. 09340 México DF, Mexico; b Unidad de Proteómica, Centro de Investigación Príncipe Felipe, C/Rambla del Saler 16, 46012 Valencia, Spain; c Departamento de Biología Celular, Centro de Investigación y de Estudios Avanzados del Instituto Politécnico Nacional (Cinvestav-IPN), Av. Instituto Politécnico Nacional 2508, Col. San Pedro Zacatenco, Gustavo A. Madero, C.P. 07360 México DF, Mexico; d AB SCIEX, 500 Old Connecticut Path, Framingham, MA 01701, USA; e Departamento de Ciencias de la Salud, Universidad Autónoma Metropolitana, Unidad Iztapalapa, Av. San Rafael Atlixco No. 186, Col. Vicentina, Iztapalapa, C.P. 09340 México DF, Mexico; f Departamento de Zoología, Escuela Nacional de Ciencias Biológicas, Instituto Politécnico Nacional, Prolongación de Carpio y Plan de Ayala s/n, Col. Santo Tomás, Miguel Hidalgo, C.P. 11340 México DF, Mexico; ^g^ Instituto de Enfermedades de la Mama, Fundación del Cáncer de Mama (FUCAM A.C.), Av. Bordo No. 100, Col. Viejo Ejido de Santa Ursula Coapa, Coyoacán, C.P. 04980 México DF, Mexico

## Abstract

Breast cancer is the most common and the leading cause of mortality in women worldwide. There is a dire necessity of the identification of novel molecules useful in diagnosis and prognosis. In this work we determined the differentially expression profiles of four breast cancer cell lines compared to a control cell line. We identified 1020 polypeptides labelled with iTRAQ with more than 95% in confidence. We analysed the common proteins in all breast cancer cell lines through IPA software (IPA core and Biomarkers). In addition, we selected the specific overexpressed and subexpressed proteins of the different molecular classes of breast cancer cell lines, and classified them according to protein class and biological process. Data in this article is related to the research article “Determination of the protein expression profiles of breast cancer cell lines by Quantitative Proteomics using iTRAQ Labelling and Tandem Mass Spectrometry” (Calderón-González et al. [1] in press).

**Specifications table**

**Table:** 

Subject area	Cell Biology
More specific subject area	Breast cancer
Type of data	Tables, figures
How data was acquired	Isobaric labelling, preparative isoelectrofocusing, reverse phase chromatography and tandem mass spectrometry using an AB SCIEX high performance hybrid quadrupole time-of-flight QSTAR ESI XL Hybrid LC/MS/MS Mass Spectrometer System
Data format	Analysed and filtered
Experimental factors	Samples were reduced with 50 mM TCEP, alkylated with 200 mM MMTS, digested with sequencing grade trypsin and labelled according to the manufacturer׳s protocol of iTRAQ 8-plex kit, with minor modifications
Experimental features	An amount of 110 μg of total protein from each normal and breast cancer cell line were trypsin-digested, iTRAQ-labelled and pooled. Labelled-peptides were separated by isoelectrofocusing on a non-linear pH 3–10 gradient. Strips were divided in sections and peptides extracted and chromatographed in a C18 reverse phase column. Elution was done using several linear gradients of ACN and eluted peptides were analysed in a QSTAR ESI XL Hybrid LC/MS/MS Mass Spectrometer System. Collected mass spectra were analysed with the 4.0 ProteinPilot program and a cut-off of 20% fold change in the relative quantitation ratio was set. Identified proteins were grouped and classified with PANTHER (v 9.0) and IPA software
Data source location	Mexico City, Mexico
Data accessibility	Data is within this article

Value of the data

• Differential protein expression profiles of breast cancer cell lines.
• Diseases and biofunctions analysis by IPA of over- and subexpressed polypeptides found in common in all breast cancer cell lines.
• Comparative analysis of putative biomarkers found in common in all breast cancer cell lines.
• Identification of specific over- and subexpressed proteins for each breast cancer cell line.
• Classification of specific proteins in each breast cancer cell lines according to protein class and biological process with PANTHER.

## 1 Data

We identified 1020 iTRAQ-labelled proteins with at least one peptide with a minimum of 95% in confidence with the ProteinPilot software (Table 1).

### 1.1 IPA analysis of the differentially expressed proteins found in common in all breast cancer cell lines

To determine whether over- or subexpressed polypeptides have been involved in diseases and biofunctions, or localised in networks, we used the IPA software to perform mainly three different core analyses: (a) Core I considered all tissues, primary cells and all cell lines. (b) Core II was performed for breast cancer cell lines and mammary gland. (c) Core III, for all cancer cell lines excluding breast cancer cell lines (Table 2). They were obtained for different categories of diseases and biofunctions, using a p-value equal or lower than 0.05, which was obtained with Fisher´s Exact Test. Core analyses I and III shared similarities in the results obtained. Core I included 78 categories of diseases and biofunctions, whilst 11 categories were found in Core II and 72 in Core III. The most representative categories in Core I were Nucleic Acid Metabolism (16 proteins and *p*-values between 4.46×10^−10^ and 2.53×10^−2^), Small Molecule Biochemistry (36 polypeptides and *p*-values ranging from 4.46×10^−10^ to 3.77×10^−2^), DNA Replication, Recombination and Repair (19 proteins, *p*-values between 7.24×10^−10^ and 3.77×10^−2^), and Energy Production (10 polypeptides and a *p*-values of 7.24×10^−10^). In Core II we got the following categories: Cellular Response to Therapeutics (only 2 proteins with a *p*-value of 2×10^−3^), Cellular Movement (9 polypeptides and *p*-values from 1.72×10^−2^ to 3.67×10^−2^), Cell Morphology and Cellular Assembly and Organisation (2 polypeptides, *p*-value of 1.85×10^−2^). Finally, the most representative categories in Core III were the same as in Core I: Nucleic Acid Metabolism (16 polypeptides, *p*-values ranging from 4.6×10^−10^ to 4.02×10^−2^), Small Molecule Biochemistry (34 proteins, *p*-values between 4.6×10^−10^ and 4.02×10^−2^), DNA Replication, Recombination and Repair (21 proteins, *p*-values from 7.46×10^−10^ to 3.78×10^−2^), and Energy Production (12 proteins, *p*-values from 7.46×10^−10^ to 4.02×10^−2)^.

**Fig. 1:**
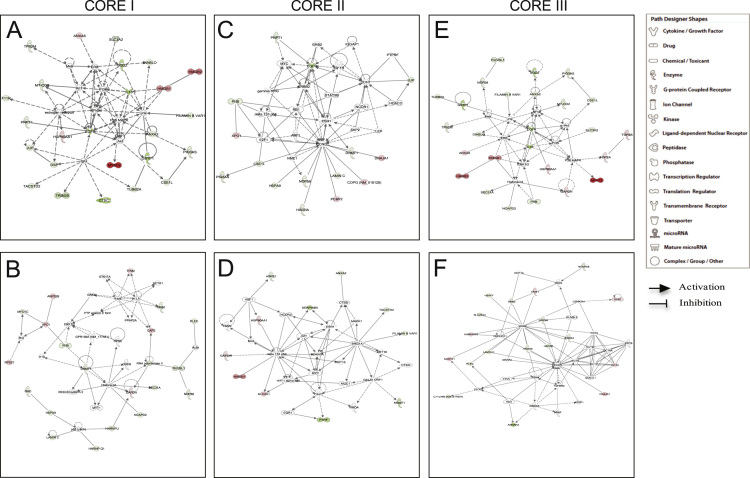
IPA functional networks of differentially expressed proteins through different core analyses. There are only shown the two top networks of each core analysis. Core I networks are related with: (A) Cancer; Reproductive System Diseases; Dermatological Diseases and Conditions; Haematological Diseases. (B) Connective Tissue Disorders; Neurological Diseases; Skeletal and Muscular Disorders; DNA replication, Recombination and Repair; Cancer; Gastrointestinal Diseases. Core II networks are implicated in: (C) Cellular Development; Cellular Growth and Proliferation; Cell Cycle. (D) Cell Death and Survival; Tumour Morphology; Cellular Development. Core III analysis was performed for proteins involved in: (E) Cell Cycle; Cancer; Hereditary Disorders; Haematological Diseases. (F) Developmental Disorders, Hereditary Disorders; Metabolic Diseases; Cell Death and Survival. Different shapes, indicating the functional class to which they belong, represent proteins. Molecules in red are overexpressed polypeptides, whilst those green molecules correspond to subexpressed proteins. Molecules in grey, which are not specified, were incorporated into the networks by IPA through relationships with other molecules. Molecular relationships between polypeptides are indicated with lines. A continuous line illustrates a direct interaction and a dotted line is used for indirect interactions.

The two top networks obtained in Core I analysis were related with: (1) Cancer; Reproductive System Disease; Dermatological Diseases and Conditions; Haematological Diseases, which showed a score of 38 and contained 25 target molecules. (2) Connective Tissue Disorders; Neurological Disease; Skeletal and Muscular Disorders; DNA Replication, Recombination and Repair; Cancer; Gastrointestinal Diseases, that had a score of 27 and a network focused on 20 molecules (Fig. 1A and B). In Core II analysis, the two most representative networks were involved in: (1) Cellular Development, Cellular Growth and Proliferation, and Cell Cycle, with a score of 15 and a network of 15 polypeptides. (2) Cell Death and Survival, Tumour Morphology, and Cellular Development. In this case, the score was 9 and the network generated was focused on 11 molecules (Fig. 1C and D). Finally, the Core III analysis contained polypeptides playing a role in: (1) Cell Cycle, Cancer, Hereditary Disorders, and Haematological Diseases. In this case, it showed a score of 41 and considered 26 proteins (Fig. 1E). (2) Developmental Disorders, Hereditary Disorders, Metabolic Diseases, and Cell Death and Survival. This network had a score of 24 and contained 18 polypeptides (Fig. 1F).

### 1.2 Selection of putative biomarkers found in common in all breast cancer cell lines

For the identification of putative biomarkers we used two different approaches: (1) Filtering candidate biomarkers with the IPA software, and (2) Searching of proteins in the PubMed database. The first filter, named Biomarkers I, was carried out in all tissues, primary cells and all cell lines information contained in the Ingenuity Knowledge database; the second one was through breast cancer cell lines and mammary gland (Biomarkers II), and the third consisted in filtering through cancer cell lines, excluding breast cancer cell lines and was named Biomarkers III. Results obtained in Biomarkers I revealed 39 potential candidates: ACAA2, ACAT1, ANXA2, CAP2, CPT1A, CTNNA1, CTSC, CYB5R3, DNMT1, EGFR, EIF4B, ETFB, F11R, FLNB, GAPDH, GLUD1, GSTP1, HADHA, HADHB, HLA-A, HMGB1, HMGB2, HNRNPU, HSP90AA1, IL18, JUP, MCM2, MYH9, NNMT, PEBP1, PHB, SERPINB5, SLC2A1, SOD2, TOP2A, TPI1, TPM2, TRIM29, XPO1. Biomarkers II included 9 putative targets: CTSC, CYB5R3, EGFR, EIF4B, FLNB, GSTP1, IL18, MYH9, TOP2A. Finally, Biomarkers III selected 24 polypeptides, which included ANXA2, CAP2, CTNNA1, DNMT1, EGFR, GAPDH, GLUD1, GSTP1, HLA-A, HMGB1, HSP90AA1, IL18, JUP, MCM2, NNMT, PHB, SERPINB5, SLC2A1, SOD2, TOP2A, TPI1, TPM2, TRIM29 and XPO1. All of these potential biomarkers were in common in all studied breast cancer cell lines.

**Fig. 2:**
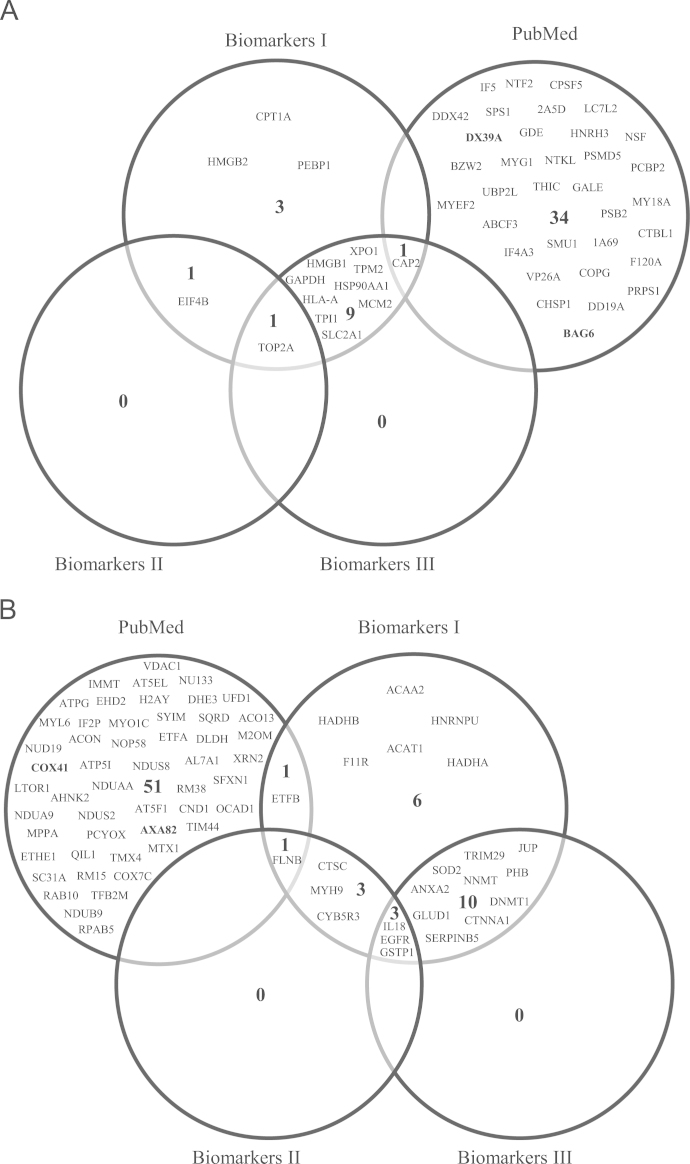
Venn-Euler diagrams of putative biomarkers obtained through IPA and PubMed analyses. Diagrams shows the putative biomarkers found with IPA Biomarkers filter I (all tissues, primary cells and all cell lines), II (mammary gland and breast cancer) and III (cancer cell lines excluding breast cancer cell lines), and those obtained with PubMed database search. (A) Overexpressed proteins and (B) Subexpressed polypeptides common in all breast cancer cell lines.

In relation to the search for candidates in the PubMed database, we obtained 35 overexpressed and 53 subexpressed polypeptides not previously reported in breast cancer, with one exception, ANXA8, which has only one report [2]. Finally, we performed a comparison between the biomarkers found with IPA and those in the PubMed search. These analyses revealed only 3 specific overexpressed polypeptides in Biomarkers I, and 34 in the PubMed search (Fig. 2A). For subexpressed proteins, we obtained 6 polypeptides for Biomarkers I, and 51 in PubMed (Fig. 2B). Biomarkers II and III modules did not find exclusive markers in both cases.

### 1.3 Panel of putative biomarkers exclusively found in Luminal A, Claudin-low and HER2^+^ breast cancer cell lines

**Fig. 3:**
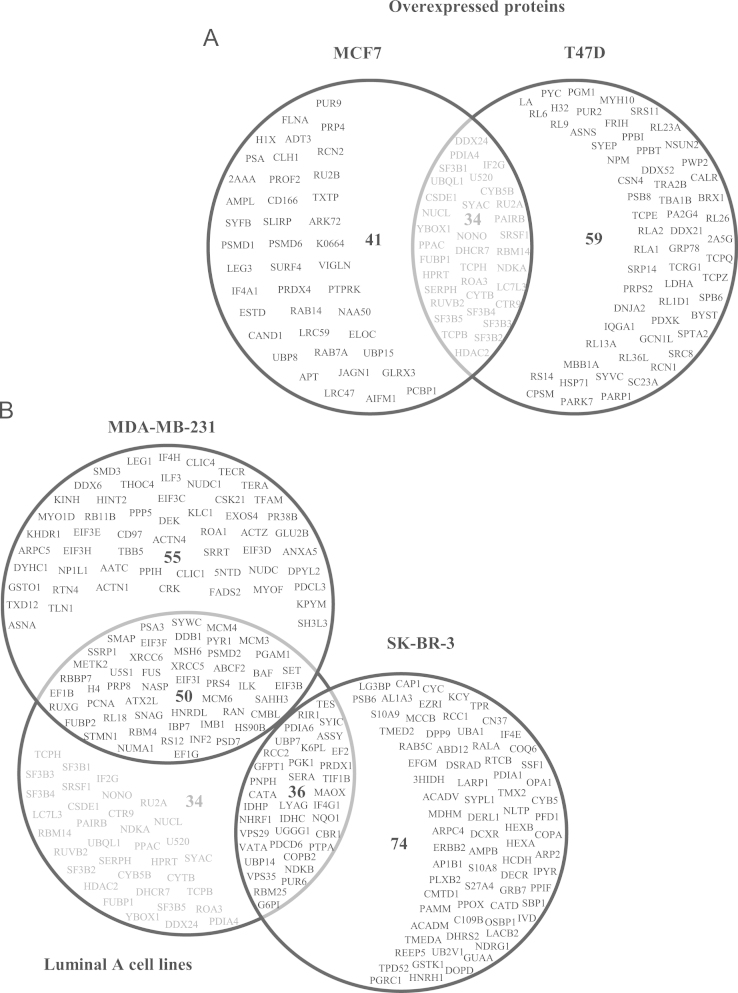
Venn-Euler diagrams of overexpressed proteins in the different molecular types of breast cancer cell lines. (A) The diagram shows the specific proteins in the Luminal A cell lines (MCF7 and T47D) as well as common proteins in these lines. (B) Diagram shows the unique proteins for Claudin-low (MDA-MB-231) and HER2^+^ (SK-BR-3) cell lines, and the comparison of the common proteins in Luminal A cell lines with the other types of cell lines.

**Fig. 4:**
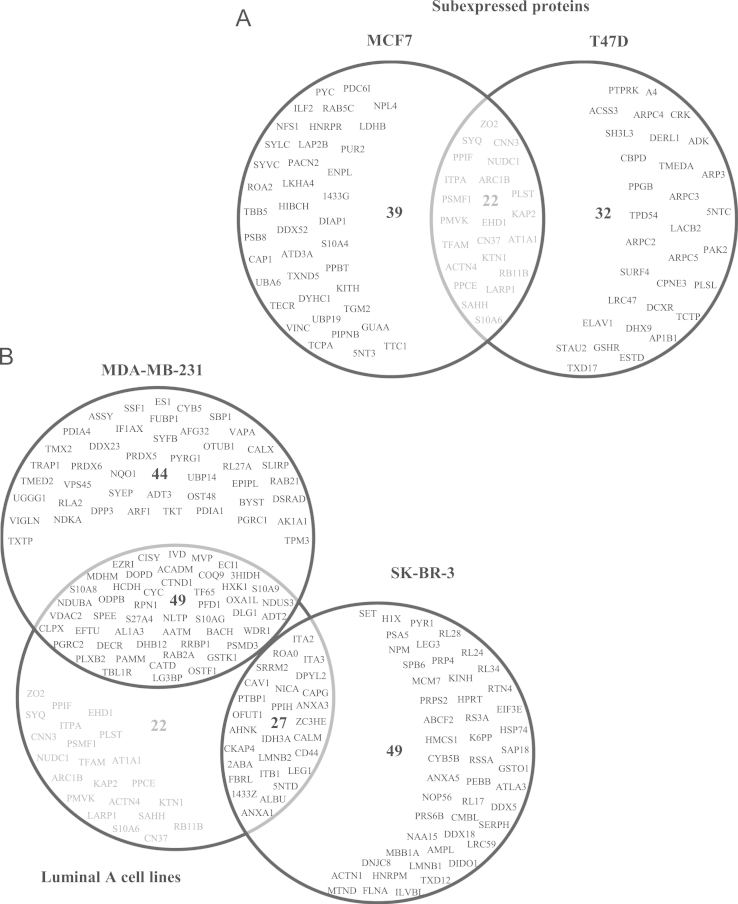
Venn-Euler diagrams of subexpressed proteins in the different molecular types of breast cancer cell lines. (A) The diagram shows the specific proteins in the Luminal A cell lines (MCF7 and T47D) as well as common proteins in these lines. (B) Diagram show the unique proteins for Claudin low (MDA-MB-231) and HER2^+^ (SK-BR-3) cell lines, and the comparison of the common proteins in Luminal A cell lines with the other types of cell lines.

We identified sets of specific proteins for Luminal A, Claudin-low and HER2^+^ breast cancer cell lines. We obtained a set of proteins shared by Luminal A cell lines containing 34 overexpressed (Fig. 3A) and 22 subexpressed polypeptides (Fig. 4A). In the case of Claudin-low, we identified a set with 55 and 44 overexpressed and subexpressed polypeptides, respectively (Figs. 3B and 4B). The HER2^+^ cell line showed 74 overexpressed (Fig. 3B) and 49 subexpressed proteins (Fig. 4B). However, we need to determine the expression levels of these proteins in tumour tissues and or sera to validate them as biomarkers.

### 1.4 Functional classification of the exclusive proteins differentially expressed in each breast cancer cell line

**Fig. 5:**
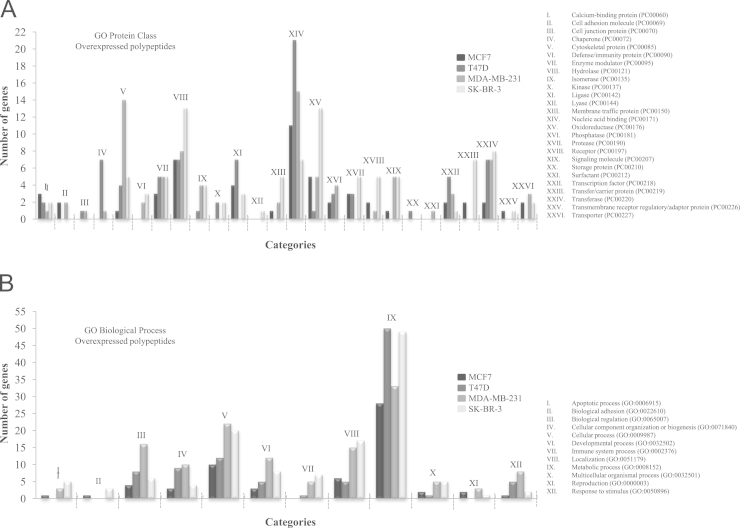
PANTHER functional classification of overexpressed proteins found exclusively in each breast cancer cell line. The graph shows the number of genes involved in the different Protein Class (A) and Biological Process (B) of the specific proteins in each breast cancer cell line. The total numbers of class hits were 54, 77, 84 and 97 for Protein Class and 61, 96, 132, and 127 for Biological Process in MCF7, T47D, MDA-MB-231 and SK-BR-3 cell lines.

**Fig. 6:**
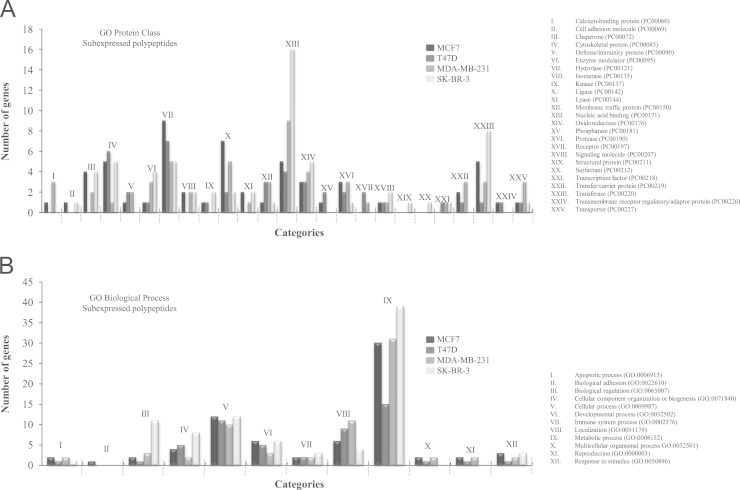
PANTHER functional classification of subexpressed proteins found in each breast cancer cell line. The graph shows the number of genes involved in the different Protein Class (A) and Biological Process (B) of the specific proteins in each breast cancer cell line. The total numbers of class hits were 57, 41, 55 and 64 for Protein Class and 72, 52, 70, and 87 for Biological Process in MCF7, T47D, MDA-MB-231 and SK-BR-3 cell lines.

The overexpressed and subexpressed specific proteins of each breast cancer cell line (MCF7, T47D, MDA-MB-231, SK-BR-3) were classified with PANTHER. For Protein Class of overexpressed proteins, we obtained 26 categories, being the Nucleic Acid Binding (XIV) the most representative category for MCF7, T47D and MDA-MB-231, and Hydrolase (VIII) and Oxidoreductase (XV) for SK-BR-3 (Fig. 5A). In the case of the Biological Processes, we found 12 categories, being the Metabolic process (IX) the category with more genes implicated (Fig. 5B). On the other hand, the categorisation of subexpressed proteins specific for each cell line had 25 categories in Protein Class and 12 in Biological Processes. The categories of Protein Class with mayor number of genes were Hydrolase (VII) for MCF7, and T47D, Nucleic Acid Binding (XIII) for MDA-MB-231 and SK-BR-3 (Fig. 6A). The category of Biological Process most affected was Metabolic process (IX) in each of cell line (Fig. 6B).

## 2 Experimental design, materials and methods

### 2.1 IPA analysis of proteins in common in all breast cancer cell lines

To identify diseases and biological functions, canonical pathways, molecular networks, and putative candidates for biomarkers from the proteins that are commonly express in all breast cancer cell lines, we used the complete functional Ingenuity Pathway Analysis software (IPA, QIAGENs Redwood City, www.qiagen.com/ingenuity). We performed three different analyses named Cores I, II and III. In all cases we used the stringent filter. The Fisher׳s Exact Test was used to determine the *p*-value, which was considered as significant with values ≤0.05. Core I was done to classify identified proteins in all tissues, primary cells and all cell lines including cervical, central nervous system, colon, hepatoma, immune, kidney, leukaemia, lung, lymphoma, macrophage, melanoma, myeloma, neuroblastoma, osteosarcoma, ovarian, pancreatic, prostate, teratocarcioma, breast cancer and other cell lines. Core II was used in mammary gland and breast cancer cell lines. Core III in all cancer cell lines, excluding breast cancer cell lines. Parameters used in the complete analysis were: (i) ingenuity knowledge base (genes only), considering direct and indirect relationships. (ii) Interaction networks including endogenous chemicals, default value of 35 molecules per network and 25 networks per analysis. (iii) All data source. (iv) Confidence: experimentally observed, highly and moderately predicted. (v) Human species with stringent filter. (vi) All mutations.

### 2.2 Selection of putative biomarkers common in all breast cancer cell lines

We used the module IPA biomarker to prioritise protein biomarker candidates in our data. Three analyses were carried out, Biomarkers I included a filter for all tissues, primary cells and all cell lines, Biomarkers II for breast cancer cell lines and mammary gland, and Biomarkers III for cancer cell lines, excluding breast cancer. Parameters used were: (1) human species. (2) All molecule types. (3) All biofluids. (4) All diseases for Biomarkers I, and cancer disease for Biomarkers II and III. (5) Biomarkers I, all biomarkers and disease application. For Biomarkers II, all biomarkers application and breast cancer disease (including breast cancer, breast carcinoma, ductal carcinoma, ductal carcinoma in situ, infiltrating ductal breast carcinoma, invasive ductal breast cancer, lobular breast carcinoma, and metastatic breast cancer). In the case of Biomarkers III, all biomarkers application and cancer disease excluding breast cancer.

Moreover, we searched every one of the 206 identified polypeptides that changed in their expression levels in common in all breast cancer cell lines in PubMed database using key words such as biomarkers, cancer and breast cancer. Finally, we performed a comparison analysis of the results obtained with IPA Biomarker modules and PubMed with the Venny program (developed by Oliveros JC and available at http://bioinfogp.cnb.csic.es/tools/venny/).

### 2.3 Selection of biomarkers panel in the different molecular classification of breast cancer cell lines

For the selection of the exclusive sets of overexpressed and subexpressed polypeptides for Luminal A, Claudin-low and HER2^+^ cell lines, we performed a comparative analysis of between them with the Venny program.

### 2.4 Functional classification of the exclusive sets of proteins for each breast cancer cell lines according to their corresponding molecular classification with PANTHER

The specific sets of polypeptides for each breast cancer cell line according to their corresponding molecular classification were analysed with the PANTHER classification system v 9.0 (http://www.pantherdb.org/) [3]
